# Involvement of frontline clinicians in healthcare technology development: Lessons learned from a ventilator project

**DOI:** 10.1007/s12553-022-00655-w

**Published:** 2022-03-11

**Authors:** Margaret Chen-Mei Lin, Tae-Ho Kim, Woo Soo Kim, Ingrid Hakanson, Ali Hussein, Lillian Hung

**Affiliations:** 1 School of Nursing, University of British Columbia, T201 2211 Wesbrook Mall, V6T 2B5 Vancouver, BC Canada; 2 Additive Manufacturing Laboratory, School of Mechatronic Systems Engineering, Simon Fraser University, Surrey, Canada; 3 Intensive Care Unit, Vancouver General Hospital, Vancouver, Canada; 4 Faculty of Science, University of British Columbia, Vancouver, Canada

**Keywords:** Co-development, Healthcare, Interdisciplinary, Collaboration, Technology, Human-factor engineering

## Abstract

Co-development of healthcare technology with users helps produce user-friendly products, ensuring safe device usage and meeting patients’ needs. For developers considering healthcare innovations, engaging user experience can reduce production time and cost while maximizing device application. The purpose of this paper is to report lessons learned from the development of a 3D printed origami ventilator prototype in response to the rise of ventilator demand due to the Coronavirus disease (COVID-19) pandemic. We conducted focus groups with frontline clinicians working in an Intensive Care Unit of a large urban hospital in Vancouver, British Columbia, Canada. In the interdisciplinary focus groups, we identified challenges, practical tips about product development, the human needs of technology, and cross-discipline peer learning. The focus group discussions provide useful insight into the technology development for complex clinical contexts. Based on our experiences, we articulate five practical tips for co-development of healthcare technology - AGILE: Analyse users’ needs first, Gain insights into complex context, Involve users early and frequently, Lead with a prototype, and Educate and support. Through sharing the tips and lessons learned, we wish to emphasize the necessity of meaningful multi-disciplinary collaboration during healthcare technology development and promote the inclusion of frontline clinicians during these initiatives.

**Supplementary Information:**

The online version contains supplementary material available at 10.1007/s12553-022-00655-w.

## Background

During the COVID-19 pandemic, technologies have been used to support healthcare capacity to meet care needs globally. Three-dimensional (3D) printed devices especially gained a lot of popularity amidst the supply shortage during the pandemic because of their efficient production, adaptability, and minimized cost and time [1]. As recommended by the literature, the involvement of healthcare providers when developing healthcare technology such as 3D printed medical devices is especially encouraged to maximize technology application and safe patient care usage [2, 3]. At the same time, however, little is known about how academic researchers, industrial developers, and frontline clinicians may collaborate and contribute to successful product development [4].

Innovation complexity in healthcare involves understanding and responding to the needs of multiple stakeholders (e.g., physicians, nurses, therapists, and patients who may have complex and diverse medical conditions) in various clinical situations (critical and non-critical care, acute and community care). Without meaningful involvement of clinician users in product design, technology developers may make misassumptions about users’ needs and miss opportunities to tailor the product development to ensure safety, usefulness, and clinical relevance. Braithwaite [5] explains that frontline staff would resist change when a new innovation is imposed by a top-down approach without considering feedback loops for learning, showing their power to resist. New technology can be wasted or not used when the design does not consider the complexity of human factors and when it is not supported by parties with the power to resist. MacNeil et al. [6] conducted a review of 67 healthcare innovation and technology development; they identified the salience of consulting clinicians early in the development phase and incorporating their feedback to ensure the innovations would work well within the health system. Tang et al. [7] reported that user involvement in their project had improved user interface design, identified software defects, created ways that facilitated workflow, and identified necessary changes to the scope of the project early on.

In our ventilator project, the engineering team and researchers collaborated with frontline clinicians in an intensive care unit via focus groups to gain feedback on a 3D printed ventilator prototype. Through the collaborative experience, we observe how all disciplines can benefit from the co-development process. In this paper, we summarize the key lessons learned from the ventilator project and offer five practical tips- AGILE, which stands for: Analyse users’ needs first, Gain insights into complex context, Involve users early and frequently, Lead with a prototype, and Educate and support. We hope that our lessons learned and practical tips are helpful for future healthcare technology development initiatives, ensuring the end-products are user-friendly, safe, efficient, and maximize the quality of care to patients and clinicians. Moreover, we wish to encourage a collaborative practice that will empower all stakeholders during healthcare technology development.

## Methods

An Engineering professor and a Ph.D. student from Simon Fraser University (SFU) led the development of the ventilator prototypes. A Nursing professor and a graduate student at the University of British Columbia (UBC) led three focus groups to provide clinical opinions, input, and feedback to support the development of the ventilator. A Respiratory Therapy Educator (RTE) helped recruit Intensive Care Unit (ICU) clinicians from an urban hospital in British Columbia, Canada. We used the convenience sampling method. A poster was posted in the Intensive Care Unit at the hospital to invite participation; a group email was sent to the frontline staff, including physicians, registered nurses (RN), registered respiratory therapists (RRT), and respiratory therapy educators. Written consent was obtained from all participants. The demographic of participants are shown in Table 1.

The focus groups were held outside work hours (in the afternoon and evenings), virtually by Zoom meetings, lasting about 45–60 min each. The focus group discussions were recorded and transcribed verbatim. Two authors who led the focus groups utilized a team approach to ensure all participants had the chance to speak during the virtual focus groups. One acted as a facilitator and another as a notetaker/observer. The notetaker monitored which participant has or has not spoken, observed unmuting actions during conversations, and informed the facilitator to engage the participants as needed. Staff participants each received a $50 Amazon gift card to compensate for their time of participation. In the focus groups, we asked: (1) What has the experience been like when caring for ventilated patients during the COVID-19 pandemic using existing ventilators? (2) What is your opinion about the new portable ventilator prototype design? (3) What can be done to improve this ventilator design? The lead engineer demonstrated the ventilator prototype virtually to the clinicians through pre-recorded video with live explanations. A paper describing the ventilator prototype’s details and development was submitted for publication [8].

**Fig. 1:**
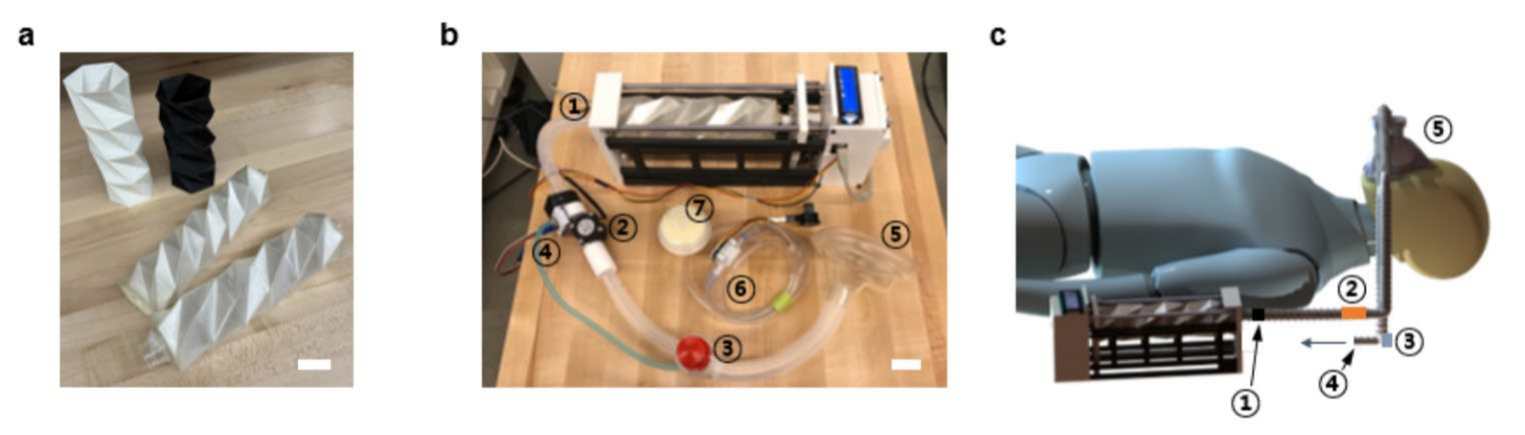
(a) Actual image of origami airtube designs (b) Actual image of the prototype: 1-A one-way valve (check valve), 2- Airflow sensor, 3-PEEP valve, 4-Pressure sensor, 5-Mask, 6-Air tube for intubation, 7-Air filter, and (c) Schematic of the breathing circuit interface between the patient and the ventilator

The three-dimensional (3D) printed ventilator uses an origami-designed airbag with a pattern of linear creases or fold lines. All of the frames are prepared by using 3D printing techniques. The fold line pattern may enable the airbag to more efficiently distribute mechanical stresses during compression cycles while helping to minimize the overall stress that is applied to the airbag. The airbag is a tuneable and sufficiently small size. When incorporated with other ventilator components, the device is easily portable with a total weight of under six kilograms, including breathing circuits. The 3D origami airbag is actuated by the linear actuator to create constant airflow. The actual image of the breathing circuit with its corresponding schematic is presented in Fig. 1. When the 3D origami airbag is compressed, air flows to the patient through a one-way valve. The airflow sensor and the pressure sensor monitor the airflow. By integrating the LCD module, the tidal volume (Vt) and respiration rate (RR) are displayed during the activation of the ventilator. The button switch and potentiometer are used for on/off activation and controls of Vt and RR, respectively.

**Table 1:** Participant Demographics

Variable	Numbers	Percentage (%)
**Gender** Male Female	7 11	38.9 61.1
**Discipline** RRT RN Physician RTE	7 5 3 3	38.9 27.8 16.7 16.7
**Years of Practice** 1–5 6–10 11+	1 10 7	5.3 52.6 38.9

## Data analysis

Focus group discussions were audio-recorded, transcribed verbatim and analysed using inductive thematic analysis [9]. First, transcripts were read several times by the UBC student in order to become familiar with the text. Second, relevant extracts were highlighted and coded. Third, these codes were organized into categories. Fourth, all authors from both universities were involved in team discussions to develop preliminary themes. The final step involved finalizing themes and selecting representative quotes to support each theme. Throughout, the analysis followed a reflexive and iterative process.

## Result

Through the focus group discussions, frontline clinician participants of different disciplines and experience levels shared their care experience with ventilated patients and provided feedback on the new ventilator prototype. The engineering team and participants exchanged different perspectives and learned from each other’s profession. They voiced appreciation for the collaboration opportunity and desire for more co-development opportunities. A table of highlighted quotes from participants can be found in supplementary file 1.

### Learning for the Engineering Team

Lessons learned by the engineering team included: clarified direction on product development, appreciation for the human factors of technology, and awareness of extra resources that would have otherwise been inaccessible or unknown to the engineering team.

#### Direction of Product Development

In the focus groups, clinician participants voiced many possibilities of ventilator usage and its target patients with the engineering team. As a result, the engineering team gained useful insight into the complexity of clinical processes and targeted use in various clinical contexts.

In focus group #1, an experienced registered respiratory therapist stated how this current ventilator design could benefit a ‘very special subset’ of ‘high-level spinal cord injury patients’ and Amyotrophic Lateral Sclerosis (ALS) patients. An anesthesiologist pointed out that:

Even people with high spinal cord injuries and most severely brain-injured patients, unless they’re in a highly monitored ICU, typically will have some capacity to interact with the ventilator. You would need to translate it to something that has some capacity to sense the flow that’s generated on the patient.

In addition to the need for sensing ability, a clinician also stated that the ventilator would need to include machine synchronicity (the ability for the machine to synchronize with the patient’s breathing pattern) before it can be used on patients:

Synchrony is a big deal… if you’re thinking that this is like disaster response, like an earthquake in the jungle, and you’ve got to get 20 people who are critically ill out of there and into an urban centre, then you can knock them out with pharmaceuticals for that transport. But if it’s something that you can add, I would add it. (Respiratory Therapy Educator, RTE)

These suggestions clarify that some improvements to the ventilator product are necessary.

*“Our development has focused more on mechanical durability and a bit less on function. Today, we have learned various important features like oxygen connection, alarming, PEEP, those kinds of things. Our portable ventilator design may be more useful for patient transport and in remote areas. (Lead engineer)”*.

The interdisciplinary focus group discussions helped the engineering team refine their project objectives and identify gaps for improvement.

In addition to identifying potential users and related concerns, the focus groups discussed necessary improvements in machine functions and the critical criteria for future ventilator development. The clinicians recommended additional features such as oxygen capacity, high and low-pressure alarm for safety, and a patient trigger (a method for the ventilator to adjust when patients wish to take extra breaths in addition to the pre-set frequency):

You need a volume waveform, pressure, and time. Or volume [vs] time, pressure [vs] time and flow [vs] time. They would all have to be in concurrent, as a picture all together per breath; that would be what you would see each time because each waveform would identify different problems. (RTE)

*“We need to have some sort of portable battery that could be utilized for running and monitoring ventilating systems. (Registered Respiratory Therapist, RRT)”*.

With suggestions on specific functions, clinicians also offered important knowledge based on their clinical experience:

Is [the air] being filtered, or is it just going straight into the atmosphere? You need to use a bacterial-viral filter because ventilated patients may have highly infectious diseases. (Physician)

I noticed your motor speed seems rather fixed. In terms of how patients get air, we need a variability of flow to determine the inhale time and exhale time. (physician)

During the discussion about necessary improvements, the engineering team had opportunities to gain a deep understanding of technical suggestions from the clinicians:

PEEP stands for Positive End-Expiratory Pressure. It’s the amount of pressure that’s left in the patient’s lungs at the end of the breath, so it helps to prevent lung collapse…think of it like a balloon, it’s really hard to start the opening of the balloon, you need a lot of pressure at first, but then when you hit that sweet spot, the balloon opens very easily, and you can easily put air in and out so that’s why PEEP is an important tool. (Physician)

While identifying and explaining the improvements needed, the clinicians emphasized the necessity for some of these improvements, classifying them as standards rather than preferred functions for ventilators. For example:

You will not sell one ventilator if you can’t give oxygen. No patient is on a ventilator unless they need a large amount of oxygen. (RRT)

Certain features that we discussed today would be more the standard. We couldn’t even start talking about using it in the field or having EHS [Emergency Health Service] playing with it at all if you didn’t have the basic modes. (RTE)

EHS, which stands for Emergency Health Services, represents paramedics and first responders that many participants mentioned when considering the design of the ventilator. Along with the necessary improvements, clinicians highlighted strengths in the new design:

There are a few things that I really like. I like the small size and its rectangular shape because when you’re thinking about an EHS bed to go to the ambulance, you need a space-saving ventilator, and it’s nice to be able to fit it between the person and the edge of the bed. Also, I like how clear the buttons are, as sometimes [with] the ventilators, you don’t know where the “on” button is since they’re behind some trap door kind of thing. Especially if you’re trying to cater to EHS, they need to know where certain buttons are, without too much fuss. (RRT)

Clinicians also provided useful resources such as links about ventilator mouthpieces, required alarms and specific ventilator models:

If your audience is the EHS, then I would probably compare your ventilator to what they currently use. Most of them use the LTV 1200 ventilator, which can do volume control, pressure control, pressure support with PEEP and oxygen capability. (RRT)

[The prototype] kind of reminds me of a transport ventilator, smaller in size. If it has a good battery, kind of like our T1 Hamilton ventilator. (RRT)

These suggestions provided the engineering team with constructive directions on how to proceed with product development.

#### Human perspective

Other than specific knowledge about ventilator functions, the engineering team learned to consider human perspectives when developing the technology. The ergonomic needs introduced to the engineers by the clinicians were derived from clinicians’ practical experience and their witnessing of patients interacting with ventilators. The clinician participants explained why these ergonomic considerations are essential, and the engineering team expressed their recognition for this new insight.

***Clinicians’ Needs.*** When asked about their experiences of taking care of patients on ventilators, clinicians expressed needs related to size, waterproof function, and alarms:

Sometimes we have to bring the patient to transport or MRI, then there’s the big clunky ventilator and IV pumps and other machines as well. Our hallways tend to be very narrow, especially the MRI department, so I like the ventilator that you guys have, which is really small and compact. I’m wondering if you can fit it onto the patient’s bed during transport and whether it’s MRI compatible as well. If you can bring it in; that would be great. (RRT)

It’s amazing how much fluid is around an intensive care patient. Staff are hanging tons of bags of fluid that when you spike the bags, they leak. Sometimes the patient can be bleeding. For patients with pulmonary edema, bloody fluid could land on top of that, so [the ventilator] would have to be impermeable to fluid. (RRT)

Clinicians in different disciplines also have specific needs. For registered respiratory therapists and registered nurses, factors that directly affect patient safety and quick operation were specially mentioned:

Being able to put the vent into standby easily is probably helpful in these situations because then when we do disconnect the patient for a reason, it’s not going to be blowing everywhere. Instead of having to turn the whole ventilator off. (RRT)

The things that they always teach nurses in critical care are where’s my silence alarm and where do I give extra oxygen if I need it. It sounds simple, but it’s like your emergency alarm. So if you are going to allow that alarm system in, make sure there’s a way to reset that alarm. It sounds simple, but it can be forgotten. The other thing is if you are going to include extra oxygen with it…maybe humidifying air as well…(RN)

For physicians, ventilators also act as a diagnostic device:

A ventilator is not just a delivery of a therapeutic device. I also use it as a diagnostic device, so the data that it gives us include lung compliance, oxygenation, ventilation pressure, volume curves… those kinds of metrics are very, very important for us to provide safe care. (Physician)

***Patients’ Needs.*** In addition to operational needs, clinicians explained why the ventilator has to respond to the patients’ needs:

Most patients are partially awake, so it’s the interactions between the patient’s needs and the ventilator’s ability to provide those needs. It’s usually partially assistant ventilation versus a complete control form of ventilation, so it really needs to be kind of interactive with how the patient sees the demands. (RRT)

Let’s say when the breathing tube gets compressed, does the ventilator just keep on pushing air? Or like when the patient has a pneumothorax, does it have a high-pressure alarm? (RRT)

Examples were given to the engineering team to emphasize the significance of machine responsiveness on patient safety:

The easiest way to think about it is if you’re going to try and put in your 600 mL [of oxygen], which fits into a big person and they’re on transport or they’re with an ambulance attendant, and their lungs start filling up with blood and your machine’s putting in 600 mL until you pop the balloon - because the balloons now only this big and it’s rock hard, and so, if your machine doesn’t have a really good pressure alarm for that volume setting, [the lung will pop]. (RTE)

It appears in the [demonstration] video that exhalation is an active process, as opposed to a passive process that we would typically see with most ventilators. It concerns me if you have active exhalation as driven by the motor, and you actually will generate negative pressures. Those negative pressures, in addition to the lack of PEEP, may further compound the problem and develop airway collapse. (Anesthesiologist)

Clinicians repeatedly mentioned the complexity of the machine-patient relationship. For example, an RRT explained:

We get a lot of patients that have large abdomens or are post-op, or they are full of fluid… PEEP tends to up those little alveoli and the lungs but helps to also splint and open the airways further…with the alarm, if I’m transporting a patient and I get a disconnect, I want to hear an alarm for circuit disconnect. I want to know that an alarm will go off. If there’s a leak in the circuit or they’re having a cuff leak in their endotracheal tube, I want to hear the alarm. For PEEP pressure, if my patient is coughing or if they’ve got secretions, I want to know if the alarm goes off.

The insights from clinicians regarding the ergonomic needs of a ventilator were a significant learning experience for both the researchers and the engineering team. The lead engineer concluded the new understanding of the human-machine relationship by stating that:

There’s so much complexity when you think about the real patient, it’s not just a machine… and we learned so much today and so much complexity when you think about the real patients who will be using the machine.

With regards to the required functions of ventilators, the lead engineer expressed how the clinicians’ perspective greatly impacted his understanding of the human-machine relationship:

As an engineer, when we think about pressure-sensing, we think about only the device’s pressure, so I always answer the audience that we have some auto-calibration methodology by ourselves, because if a device is delivering over volume, then it’s going to tune by itself to come back to regular. If a device is not delivering much, we expected the device would come up. That’s our solution before. But I heard a lot from you now, that the pressure and sensitivity you’re talking about, is about the patient. So that’s a different perspective…without discussion like this, I have no idea. (Lead engineer)

### Learning for Healthcare Clinicians

The engineering team was not the only party that gained new perspectives from the group discussion. The diverse clinician group of registered respiratory therapists, respiratory therapy educators, physicians, and registered nurses with related yet different areas of expertise created an environment where clinicians learned from their peers and the general participatory experience.

#### Peer-learning

Many clinicians showed appreciation when hearing each other’s comments during the discussion: ‘there is so much experience, she has so much knowledge, and I’ve learned from just listening’ (RRT). An RN also expressed her appreciation towards the perspective of an attending RRT by stating that she ‘agrees with the very experienced RRTs’ before proceeding to provide ‘a nursing perspective’ of her own. An RRT also expressed her amazement at the diversity of experience within the focus group:

We have a good range of teams at this meeting right now. We have [an RTE] who’s in charge and has a vast amount of experience, [an RRT] is pretty seasoned, and I’m a new grad, so I definitely appreciate hearing and comparing my perspective with the others. (RRT)

Some clinicians valued learning about the engineering team’s innovation, stating that:

This pandemic has really exposed the vulnerability of certain environments, more so than ever in the past. The idea of being able to make a ventilator on a 3D printer is kind of blowing my mind. (RRT)

#### Learning how to be an active participant

The focus group provided an opportunity for clinicians to practice being active participants in technology development, where they teach and reflect on their experiences. There were many occasions where clinicians taught the engineering team and each other about specialized knowledge:

For ventilation, we measure pressure by the centimetres of water. When I said 5 to 15 centimetres of water, that’s the pressure at the end of expiration [PEEP]. For PEEP, we usually have a circuit pressure of between 5 to 15 centimetres of water. When the ventilator delivers a breath, the pressure goes up and for a variety of reasons, if the pressure goes too high, it can result in injury of the lung, the alveoli can tear and rip. (Physician)

#### Appreciation of inclusion

Throughout the focus groups, all clinicians, engineers, and researchers of this study expressed their appreciation for this collaboration experience. Clinicians especially appreciated the opportunity to reflect, learn, and be involved. An example can be seen when an RRT says:

Thank you for bringing this project to us. I also learned a lot today and I haven’t actually thought about a lot of these things in a really long time. So it was a really interesting perspective for me to explore what we do and how we do it and how the ventilators allow us to do that, so thank you, I think it’s an excellent project.

Some clinicians stated that they most appreciated the opportunity to participate in co-developing the technology:

Thank you for including us at the front line. Everybody here on this call can tell you that [other medical technology innovators] make new hospital beds, that you can honestly tell they never asked a bedside nurse if this bed works for the patient. (RTE)

Thank you for willing to ask these questions and touch base with people that are in this field for opinions. We really value the work that you do. This is the reason why we have new technology coming in, and we have an ever-changing innovative field, so I thank you for creating this opportunity for all of us to talk. (RRT)

## Discussion and Implications

Through the focus groups, we observed learning from both the engineering team and frontline clinicians. As a result of engineer-clinician collaboration, the engineering team gained a more concrete plan for product development and a deeper understanding of the ergonomic factors for a ventilator. For the frontline clinicians, there were opportunities to reflect, learn from their colleagues and the participatory experience as a whole. To encourage this co-development and participatory practice for future developers, clinicians, and researchers, we propose AGILE – 5 practical tips based on our lessons learned. The AGILE acronym is grounded by our empirical data via the focus groups, with our thinking informed by the Agile methodology used in the project developments. The AGILE acronym approach is similar to the Agile methodology in the iterative feedback process and continuous collaboration between users and developers. AGILE suggests that when developing healthcare technology, it is vital to: Analyse users’ needs first, Gain insights into complex context, Involve early and frequently, Lead with a prototype, and Educate and support.

### A: Analyse users’ needs first

In this project, the ventilator prototype was created to assist the shortage of ventilators during COVID-19 using a 3D-printed origami structure. When the engineering team created the ventilator prototype, unfavourable outcomes were prevented by having an informative discussion with clinicians regarding ventilator safety standards and concepts. As suggested in human-factor engineering, technology efficiency is maximized when a developing team takes time to analyse user needs, required operation tasks, interface design, and the cognitive workload of users [10]. A comprehensive analysis is especially important for medical device development. The United States Food and Drug Administration (FDA) advises that assessment regarding device users, device use environments, and device user interface while developing medical devices such as ventilators is vital in ensuring device safety and product usability [11]. Whether through user engagement, literature research, or guidelines, early and continuous analysis of user needs and existing standards of an innovative idea is necessary for successful healthcare technology development.

### G: Gain insights into complex context

Nakarada-Kordic et al. [12] summarize the idea that ‘designing for health’ requires ‘understanding and empathizing with the human,’ especially with those who are providing the services, products, and interventions. Developing healthcare technology especially requires the collaboration of users because healthcare clinicians can provide insights into the complex context of healthcare, such as patient population, infection control requirements, and hospital operation processes. The valuable knowledge ensures the technology can be appropriately adapted to the environment in which they are implemented and meet the needs of both patients and clinicians [2]. In our project, the experienced healthcare clinicians indeed provided these invaluable insights ranging from device standards to environment-specific needs, which could only be made known to the engineering team through user engagement.

When involving users, it is important to ensure that diversity is encouraged as much as possible. Users of various disciplines, experience levels, demographics, and mindsets, should be involved [3]. Since our focus groups included respiratory therapists, nurses, physicians, and educators of various experience levels, tailored insights were shared from different perspectives. Including a diverse group of users not only provides the development team with holistic user feedback but also balances power relationships between all stakeholders [3, 13]. A noted barrier that prevents collaboration between industry specialists, such as engineers and clinicians, is the belief that one profession is superior to the others [4]. Through communication opportunities such as focus groups or participatory-design research that engage stakeholders of diverse experiences, specialists can understand each other’s perspectives and values, leading to improved product and care quality [4]. Our clinician participants appreciated being involved in this multi-disciplinary collaboration. It provided them with the opportunity to contribute to the development of a frequently used device actively. The engineering team appreciated the involvement of clinicians as they provided invaluable knowledge that can improve the innovation.

### I: Involve early and frequently

Though involving users, medical device developers often only engage users at later stages of the development, asking the user for usability tests and evaluation after technology production [14]. Late engagement means involved users can only make limited suggestions on items such as labels, training, or related documents, instead of sharing significant insights that can improve device usability and ergonomics [15, 16]. Users also risk becoming a ‘token’ where they are involved in developments only so the developer can pass certain qualification criteria. In our project, the engineering team engaged with frontline clinicians relatively early in the ventilator development process. This early engagement saves the development team’s cost, time, and effort, reducing the need to modify the finalized product [11, 12]. With the clinicians’ feedback early and throughout the development, the engineering team can produce the most applicable and user-friendly ventilators.

### L: Lead with a prototype

The use of prototypes early and throughout the co-development process has been shown to encourage focused discussions, help users make sense of an ambiguous innovative idea, ensure easier exploring of possibilities, facilitate Learning, and encourage empathy [13, 17]. We agree with these benefits and encourage future co-development projects to incorporate prototypes during the collaboration, as we have witnessed its benefit through our focus groups. In our project, the prototype was made by the engineering team. Due to infection control concerns during the pandemic, the prototype was demonstrated virtually and not provided to individual clinician participants to physically examine. After watching how the ventilator prototype operates, the clinicians provided feedback and suggestions on its size, compression speed, material, structure, and output data. The feedback process was smooth and to the point because of the presence of a ventilator prototype presentation in the focus groups, helping clinicians visualize and brainstorm different possibilities. A possible improvement we encourage future co-development projects to do is to engage the users in creating the prototype together. Though not performed in our project, literature shows that creating a prototype with the users together early and throughout the collaboration helps merge users’ insight and the developers’ knowledge, creating preferable products and outcomes [17–19].

### E: Educate and support

The first four AGILE considerations recommended so far are targeted toward individual development project stakeholders. These practices, however, cannot reach their full potential and adaptation without the fifth consideration: Educate and support. This fifth consideration requires systemic support from academic institutions and healthcare organizations. To educate, academic institutions such as universities can incorporate co-designing concepts into the curriculum to teach engineering students about human-factor engineering and empathic design and teach healthcare students about the importance of multi-disciplinary collaboration and active participation in improvement processes [2, 13]. To support working professionals, healthcare organizations need to develop a support system that encourages collaboration between end-users (clinicians, patients, families) and developers such as engineers [2, 20]. Some recommendations to healthcare institutes include: provide a transparent platform where developers and healthcare users can communicate without fear of disrespect, support clinicians’ additional time spent in co-development, and allow a diverse group of stakeholders without exclusion [2, 4, 13, 20, 21].

### Limitations and Implications

There were some limitations to our research. One of the limitations includes only having one method of data collection. This method used a virtual platform due to the effects of the COVID-19 pandemic, which is not as ideal as face-to-face interaction and hands-on testing, especially when discussing aspects such as product prototypes. Even though our project took place relatively early in the product development phase, the prototype was already created before engaging clinicians. For the future, we recommend involving clinicians even earlies and throughout all stages of product development. More insights can be introduced if carrying out these focus groups during iterative cycles of design and redesign in product development. Clinicians’ involvement in the design process should be supported by leadership endorsement (i.e., offering protected time and compensation). Future studies should consider expanding methods to investigate best practices for facilitating transdisciplinary research, including academics, clinicians and industrial partners. It is also important to identify barriers to effective collaboration among healthcare professionals, academic and industrial partners.

## Conclusions

Our analysis of lessons learned in the portable origami ventilator project contributes to new knowledge, such as the AGILE practical tips, that helps to promote meaningful involvement of frontline clinicians for healthcare technology development. The inclusion of the perspectives of physicians and interdisciplinary clinicians demonstrates recognition of knowledge in practice and clinical context. The practice wisdom and experiential knowledge from clinicians help produce valuable knowledge that informs product development and improvement. Notably, the study demonstrates that it is necessary and feasible to include frontline clinicians in rapid prototype development.

## Electronic Supplementary Material

Below is the link to the electronic supplementary material.

Supplementary Material 1

## Data Availability

(data transparency)
