# Supplementary material for: Involvement of frontline clinicians in healthcare technology development: Lessons learned from a ventilator project
Source: Health Technol (Berl). 2022 Mar 11;12(2):597–606. doi: 10.1007/s12553-022-00655-w (PMC8916792; doi:10.1007/s12553-022-00655-w)
Supplement: Supplementary file 1 — Supplementary Material 1 [file 12553_2022_655_MOESM1_ESM.docx]

**Supplementary File 1**

**Article Title: Involvement of frontline clinicians in healthcare technology development: Lessons learned from a ventilator project**

**Journal Title: Health and Technology**

**Highlighted Quotes from Focus Group Participants**

| **Theme** | **Subtheme** | **Quotes** |
| --- | --- | --- |
| 4.1 Learning for the Engineering Team | 4.1.1 Direction of Product Development | *“Even people with high spinal cord injuries and most severely brain-injured patients, unless they’re in a highly monitored ICU, typically will have some capacity to interact with the ventilator. You would need to translate it to something that has some capacity to sense the flow that’s generated on the patient. (Anaesthesiologist)”*  *“Synchrony is a big deal… if you’re thinking that this is like disaster response, like an earthquake in the jungle, and you’ve got to get 20 people who are critically ill out of there and into an urban centre, then you can knock them out with pharmaceuticals for that transport. But if it’s something that you can add, I would add it. (Respiratory Therapy Educator, RTE)”*  *“Our development has focused more on mechanical durability and a bit less on function. Today, we have learned various important features like oxygen connection, alarming, PEEP, those kinds of things. Our portable ventilator design may be more useful for patient transport and in remote areas. (Lead Engineer)”*  *“If you’re going to have a monitoring screen, I would also suggest having an exhale tidal volume versus an inhale tidal volume, to be able to monitor how much is going in versus how much you’re getting back. (Physician)”*  *“You need a volume waveform, pressure, and time. Or volume [vs] time, pressure [vs] time and flow [vs] time. They would all have to be in concurrent, as a picture all together per breath; that would be what you would see each time because each waveform would identify different problems. (RTE)”*  *“We need to have some sort of portable battery that could be utilized for running and monitoring ventilating systems. (Respiratory Therapist, RRT)”*  *“Is [the air] being filtered, or is it just going straight into the atmosphere? You need to use a bacterial-viral filter because ventilated patients may have highly infectious diseases. (Physician)”*  *“Your ventilator has a lot of open spots that would be challenging to clean. (RRT)”*  *“I noticed your motor speed seems rather fixed. In terms of how patients get air, we need a variability of flow to determine the inhale time and exhale time. (Physician)”*  *“PEEP stands for Positive End-Expiratory Pressure. It’s the amount of pressure that’s left in the patient’s lungs at the end of the breath, so it helps to prevent lung collapse…think of it like a balloon, it’s really hard to start the opening of the balloon, you need a lot of pressure at first, but then when you hit that sweet spot, the balloon opens very easily, and you can easily put air in and out so that’s why PEEP is an important tool. (Physician)”*  *You will not sell one ventilator if you can’t give oxygen. No patient is on a ventilator unless they need a large amount of oxygen. (RRT)”*  *“Certain features that we discussed today would be more the standard. We couldn’t even start talking about using it in the field or having EHS playing with it at all if you didn’t have the basic modes. (RTE)”*  *“This ventilator would need additional features to satisfy an awake person. (Physician)”*  *“There are a few things that I really like. I like the small size and its rectangular shape because when you’re thinking about an EHS bed to go to the ambulance, you need a space-saving ventilator, and it’s nice to be able to fit it between the person and the edge of the bed. Also, I like how clear the buttons are, as sometimes [with] the ventilators, you don’t know where the “on” button is since they’re behind some trap door kind of thing. Especially if you’re trying to cater to EHS, they need to know where certain buttons are, without too much fuss.” –RRT*  *“If your audience is the EHS, then I would probably compare your ventilator to what they currently use. Most of them use the LTV 1200 ventilator, which can do volume control, pressure control, pressure support with PEEP and oxygen capability. (RRT)”*  *“[The prototype] kind of reminds me of a transport ventilator, smaller in size. If it has a good battery, kind of like our T1 Hamilton ventilator. (RRT)”* |
|  | 4.1.2 Human Perspective:  Clinicians’ Needs | *“It does end up cluttering the room quite a bit because sometimes it’s not just a ventilator. You’ve got the nitric there, the nurses have all their pumps there. So it can be difficult at times to work with such a big ventilator in the room. (RRT)”*  *“Sometimes we have to bring the patient to transport or MRI, then there’s the big clunky ventilator and IV pumps and other machines as well. Our hallways tend to be very narrow, especially the MRI department, so I like the ventilator that you guys have, which is really small and compact. I’m wondering if you can fit it onto the patient’s bed during transport and whether it’s MRI compatible as well. If you can bring it in; that would be great. (RRT)”*  *“It’s amazing how much fluid is around an intensive care patient. Staff are hanging tons of bags of fluid that when you spike the bags, they leak. Sometimes the patient can be bleeding. For patients with pulmonary edema, bloody fluid could land on top of that, so [the ventilator] would have to be impermeable to fluid. (RRT)”*  *“The things that they always teach nurses in critical care are where’s my silence alarm and where do I give extra oxygen if I need it. It sounds simple, but it’s like your emergency alarm. So if you are going to allow that alarm system in, make sure there’s a way to reset that alarm. It sounds simple, but it can be forgotten. The other thing is if you are going to include extra oxygen with it…maybe humidifying air as well. Because the way that we need to intrinsically find oxygen is to have it humidified in some way. (Registered Nurse, RN)”*  *“O_2_ boost is really important to us because we suction; we do just as much suctioning as the RTs do, and that’s a lot of the time when the nurses or RTs, even EHS, are troubleshooting. (RN)”*  *“The top three things that we like to see is a really good monitor - because it is the number one thing that we respond to most of the time, and our patients’ alarms right, and we have to see and assess an alarm pretty quickly because our patients are so critically ill and so to have a good monitor and a good alarm system where you can sort of assess what the alarm is and understand what it means quickly. (RN)”*  *“A ventilator is not just a delivery of a therapeutic device. I also use it as a diagnostic device, so the data that it gives us include lung compliance, oxygenation, ventilation pressure, volume curves… those kinds of metrics are very, very important for us to provide safe care. (Physician)”* |
|  | 4.1.2 Human Perspective:  Patients’  Needs | *“Most patients are partially awake, so it’s the interactions between the patient’s needs and the ventilator’s ability to provide those needs. It’s usually partially assistant ventilation versus a complete control form of ventilation, so it really needs to be kind of interactive with how the patient sees the demands. (RRT)”*  *“Let’s say when the breathing tube gets compressed, does the ventilator just keep on pushing air? Or like when the patient has a pneumothorax, does it have a high-pressure alarm? (RRT)”*  *“The easiest way to think about it is if you’re going to try and put in your 600 mL, which fits into a big person and they’re on transport or they’re with an ambulance attendant, and their lungs start filling up with blood and your machine’s putting in 600 mL until you pop the balloon - because the balloons now only this big and it’s rock hard, and so, if your machine doesn’t have a really good pressure alarm for that volume setting, [the lung will pop]. (RTE)”*  *“It appears in the [demonstration] video that exhalation is an active process, as opposed to a passive process that we would typically see with most ventilators. It concerns me if you have active exhalation as driven by the motor, and you actually will generate negative pressures. Those negative pressures, in addition to the lack of PEEP, may further compound the problem and develop airway collapse. (Anaesthesiologist)”*  *“We get a lot of patients that have large abdomens or are post-op, or they are full of fluid… PEEP tends to up those little alveoli and the lungs but helps to also splint and open the airways further…with the alarm, if I’m transporting a patient and I get a disconnect, I want to hear an alarm for circuit disconnect. I want to know that an alarm will go off. If there’s a leak in the circuit or they’re having a cuff leak in their endotracheal tube, I want to hear the alarm. For PEEP pressure, if my patient is coughing or if they’ve got secretions, I want to know if the alarm goes off. (RRT)”*  *“There’s so much complexity when you think about the real patient, it’s not just a machine… and we learned so much today and so much complexity when you think about the real patients who will be using the machine. (Lead Engineer)”* |
| 4.2 Learning for Healthcare Clinicians | 4.2.1 Peer Learning | *“We have a good range of teams at this meeting right now. We have [an RTE] who’s in charge and has a vast amount of experience, [an RRT] is pretty seasoned, and I’m a new grad, so I definitely appreciate hearing and comparing my perspective with the others. (RRT)”*  Some clinicians valued learning about the engineering team’s innovation, stating that:  *“This pandemic has really exposed the vulnerability of certain environments, more so than ever in the past. The idea of being able to make a ventilator on a 3D printer is kind of blowing my mind. (RRT)”* |
|  | 4.2.2 Learning how to be an active participant | *“For ventilation, we measure pressure by the centimetres of water. When I said 5 to 15 centimetres of water, that’s the pressure at the end of expiration [PEEP]. For PEEP, we usually have a circuit pressure of between 5 to 15 centimetres of water. When the ventilator delivers a breath, the pressure goes up and for a variety of reasons, if the pressure goes too high, it can result in injury of the lung, the alveoli can tear and rip. (Physician)”* |
|  | 4.2.3 Appreciation of Inclusion | *“Thank you for bringing this project to us. I also learned a lot today and I haven’t actually thought about a lot of these things in a really long time. So it was a really interesting perspective for me to explore what we do and how we do it and how the ventilators allow us to do that, so thank you, I think it’s an excellent project. (RRT)”*  *“Thank you for including us at the front line. Everybody here on this call can tell you that [other medical technology innovators] make new hospital beds, that you can honestly tell they never asked a bedside nurse if this bed works for the patient. (RTE)”*  *“Thank you for willing to ask these questions and touch base with people that are in this field for opinions. We really value the work that you do. This is the reason why we have new technology coming in, and we have an ever-changing innovative field, so I thank you for creating this opportunity for all of us to talk. (RRT)”* |
